# Physiotherapy Management in Endometriosis

**DOI:** 10.3390/ijerph192316148

**Published:** 2022-12-02

**Authors:** Małgorzata Wójcik, Renata Szczepaniak, Katarzyna Placek

**Affiliations:** 1 Department of Physiotherapy, Faculty of Physical Culture in Gorzow Wielkopolski, Poznan University of Physical Education, 61-871 Poznan, Poland; 2 Pabianice Medical Center, Department of Physiotherapy, WSB University, 41-300 Dabrowa Gornicza, Poland; 3 Clinic and Department of Obstetrics, Women’s Diseases and Oncological Gynecology, Collegium Medicum in Bydgoszcz, Nicolaus Copernicus University, University Hospital No. 2 Jana Biziela in Bydgoszcz, 85-067 Bydgoszcz, Poland

**Keywords:** endometriosis, physiotherapy, kinesiotherapy, manual therapy, physical medicine, balneotherapy, hydrotherapy

## Abstract

Endometriosis is a disease whose underlying cause is the growth of the endometrium outside the uterine cavity. The disease is characterised by unpleasant pain in the pelvic region, irrespective of the phase of the woman’s cycle. Physiotherapy in its various forms can be an excellent complement to the gynaecological treatment of endometriosis, by virtue of reducing inflammation, alleviating pain and thus significantly improving women’s quality of life. Physiotherapy in endometriosis should include kinesiotherapy, manual therapy including visceral therapy, physical therapy, spa treatment including balneotherapy, and hydrotherapy. The aim of this study is to present the use of physiotherapy as an adjunct therapy in the treatment of endometriosis. A review of the available literature in the Medline, PubMed and Google Scholar databases was performed without being limited by the time frame of available publications on the forms of physiotherapy used in the treatment of endometriosis.

## 1. Introduction

Endometriosis describes the presence of endometrial tissue outside the natural site in which it occurs. It can take the form of small foci in the peritoneal cavity, ovaries, abdominal organs or bladder. The foci can also be located in scars from perineal incisions or Caesarean sections and grow into the muscular wall of the uterus (adenomyosis). The whole process is subject to cyclical hormonal changes, therefore in the first phase of the cycle the foci enlarge then undergo ischemic transformation during the menstrual period. The inability to expel the cells triggers an inflammatory response in the organism, in this area, and in the formation of adhesions. In addition to severe pain, endometriosis is also a risk factor for neoplasia, including endometrial or clear cell carcinoma that arise more commonly within endometriotic lesions [1].

Certain factors may increase the risk of endometriosis. These include: a family history, particularly among first-degree relatives (which conveys a 6–7-fold higher risk of endometriosis) [2], prematurity [3], low birth weight and abnormal uterine bleeding during the neonatal period [4], formula feeding of newborns [3], reduced growth during childhood [5], childhood abuse [6], painful menarche affecting social life; pain-related school absences, unsatisfactory response to non-steroidal anti-inflammatory drugs (NSAIDs) [7], migraines [8], low body mass index, pigmented skin lesions, freckles [9], infertility [10], cyclic pain that increases during menstruation [11], pain during menstruation from digestive or urological systems, diaphragm, lungs, or sciatica [12], fatigue syndrome: pain, insomnia, depression, and stress at work [13], obstetric history: miscarriages, adverse pregnancy outcomes [14,15], pelvic surgery for endometriosis or other gynaecological indications [16,17], and autoimmune diseases [18].

Women experience pain related to endometriosis, depending on the location, during intercourse, defecation or micturition. During menstruation, this pain may be exacerbated, which is associated with hormonal changes [19].

Pharmacological treatment includes hormonal therapy and analgesic treatment. Hormonal drugs lead to suppression of ovarian function and atrophy of the ectopic endometrial foci. The most commonly used hormonal preparations include danazol, gonadoliberin analogues, progestogens, oestrogen-progesterone preparations, levonorgestrel-releasing intrauterine device, gestrinone, aromatase inhibitors, and selective progesterone receptor modulators [20].

Non-medical endometriosis treatments include antioxidants [21], Chinese herbal medicines [22,23], acupuncture [24], reflexology, homeopathy, psychotherapy, exercise and sports and nerve blocks such as a superior hypogastric plexus block [25].

The aim of this study is to present the use of physiotherapy as an adjunct therapy in the treatment of endometriosis. In this article, the authors would like to draw attention to the fact that physiotherapy in its various forms can be a good complementary treatment for women suffering from endometriosis. It can reduce pain and thus improve their quality of life. For this reason, the authors decided to conduct a literature review on this topic.

## 2. Methods

A review of the available literature in the Medline, PubMed and Google Scholar databases was carried out without being limited by the time frame of available publications on forms of physiotherapy used in the treatment of endometriosis. The search was guided by the following MeSH dictionary entries related to physical medicine: endometriosis, gynaecology, physiotherapy, balneology, kinesiotherapy, massage, scar, physical activity, visceral therapy, myofascial trigger points, and lymphatic oedema.

## 3. Physiotherapy in Endometriosis

Physiotherapy in endometriosis focuses on different areas of work with patients: pre-operative physiotherapy, post-operative physiotherapy, scar therapy, and physiotherapy concentrating on pelvic floor work (urogynaecological physiotherapy). Physiotherapy for patients with endometriosis mainly focuses on kinesiotherapy, physical therapy and balneology, as well as the use of manual therapy targeting the lumbo-pelvic area, and visceral therapy. Physical activity undertaken by women with endometriosis, learning self-therapy and self-relaxation are also very important.

Kinesiotherapy will be based on working with the musculoskeletal system of the reproductive organs and using massage, which will include the pelvic area [26,27]. The use of physiotherapy in women with endometriosis has a conservative and complementary effect on the gynaecological, pharmacological and surgical treatment process. Treatment with physical methods can be an effective alternative to other forms of treatment. Physiotherapy for patients with endometriosis is a complex therapy and should have a systemic effect [28].

The therapy should take place both in the physiotherapist’s office and be augmented by spa treatment.

During the course of spa treatment, mainly natural methods are used: balneotherapy, hydrotherapy and climatotherapy. The most commonly used balneological treatments are therapeutic brine baths, sulphide and hydrogen sulphide baths, and gas and water carbonic acid baths [29]. Radon water baths are also used in spa physical therapy, depending on the availability of these waters in the spa [29,30].

Crenotherapy, a drinking treatment with therapeutic waters, is successfully used as an adjunct to systemic balneological treatment. Mud treatments are not used in women with endometriosis due to the presence of oestrogens in its composition.

Treatments with medicinal waters are used in the form of baths, irrigations and crenotherapy [31,32]. Brine baths are the most commonly used; due to their strong stimulus, local effects are obtained: improvement in microcirculation in the skin and subcutaneous tissue and stimulation of nerve endings of the autonomic system; serial performance reduces the stimulation of the autonomic sympathetic system and accelerates cell metabolism [33]. Brine baths involve the total or partial immersion of the body in water, in which sodium chloride, calcium, magnesium, potassium, iodine, bromine and other elements have been dissolved. Bathing can take place in a pool or bathtub with a water temperature of between 34° and 40° Celsius, and a brine concentration of 3–5%, respectively. During the first treatment, the patient remains in the water for 10 min, and each subsequent bath is prolonged by two or three minutes. In the series, 10–12 treatments are carried out every one or two days. A standard treatment lasts 15–20 min. While bathing, it is recommended to move the upper and lower limbs around in the water. The classic bath can be replaced by gymnastics in the water. After coming out of the water, patients should neither rinse their body nor towel themselves off. Instead, they should allow their skin to dry, as this will leave their skin coated with a layer of salt called ‘a salt mantle. The recommended rest after bathing should last at least half an hour. Brine baths have many healing properties. The chloride and sodium ions contained in the water have a beneficial effect on the functioning of the nervous system. By acting on the sensory receptors, these ions lead to a decrease in nervous excitability and an improvement in the regulation of the autonomic nervous system. Consequently, they also support the function of the internal organs. The increase in body temperature after bathing leads to an improvement in peripheral circulation, tissue oxygenation, increased diuresis and accelerated metabolism. Muscle relaxation occurs due to the exposure of the body to hydrostatic pressure. Brine baths are contraindicated in ovarian insufficiency, developmental disorders of the ovaries and pregnancy [34].

The kinds of physical therapy used in women with endometriosis are mainly light therapy, laser therapy, electrotherapy and magnetotherapy [35,36,37,38,39,40] (Table 1). Physical therapy treatments complement the comprehensive therapy of endometriosis treatment.

In the field of phototherapy, infrared, red, ultraviolet and visible rays are used. Their main action is to accelerate the absorption of exudates, and to improve blood circulation and regeneration after surgery [41]. The use of laser biostimulation aims to accelerate tissue healing and regeneration, improve microcirculation in the wound area, and accelerate the growth of fibroblasts, collagen or nerve fibres. Using low-energy laser radiation, an analgesic and anti-inflammatory effect is also achieved [42]. The use of laser therapy in the treatment of endometriosis is also based on the use of far-infrared Low-Level Laser (Light) Therapy (LLLT). These treatments have the effect of deeply regenerating and restoring tissue function. Studies show that deep infrared action is capable of increasing the proliferation and functional (functional) cellular capacity of the endometrium [41,42,43]. The effect of laser irradiation at the cellular level manifests itself as: increased production of adenosine triphosphate (ATP), increased activity of membrane enzymes, increased synthesis of deoxyribonucleic acid (DNA) and ribonucleic acid (RNA), and accelerated electrolyte exchange between the cell and its environment. At the tissue level, there is an acceleration of blood and lymph circulation, a decrease in intracapillary pressure, an increase in the excitability threshold of nerve endings and stimulation of the immune system [44,45,46].

The application of an electromagnetic field generates endogenous heat, i.e., internal overheating of the tissues. The treatment is performed on the lower abdominal area. The method by which the treatment is carried out, the duration and number of treatments in a series are individually adapted to the patient’s condition and the severity of the symptoms. It is used in the treatment of chronic conditions, where deep overheating and hyperaemia are indicated, and therefore should not be used in women of childbearing age. In this group of patients, it is advisable to use a pulse wave, as a similar effect is obtained with almost complete elimination of the heat factor [47].

The physiotherapeutic management of patients with endometriosis also includes electrotherapy, which has an analgesic and hyperemic effect. Medium- and low-frequency currents are used [36,37]. Both interference currents and Transcutaneous Electrical Nerve Stimulation (TENS) have the effect of reducing pain in the pelvic area.

Kinesiotherapy for patients with endometriosis is an important part of the treatment and involves the selection of an appropriate exercise programme with individually selected loads. Appropriately selected exercises in the post-operative period and during the treatment of inflammation have a significant effect on the patients’ recovery and functioning. Kinesiotherapy is also suitable for patients whose health status rules out surgical treatment.

Physiotherapy is also provided in the recovery room. Immobilisation after surgery can contribute to circulatory disorders and peripheral venous blood retention and increase the risk of thromboembolic and respiratory complications [48,49]. It is important that the standard of physiotherapy management is for the patient to adopt an upright position as soon as possible, and to gradually become mobile, initially at the bedside and then within the patient’s room. The point at which the patient may stand upright and at which physiotherapy may be initiated in patients having undergone gynaecological surgery is established according to medical indications and also the individual condition of the patient, her age, existing additional conditions or possible complications following surgery.

If surgery is planned then, prior to surgery, the patient is taught general conditioning exercises to prevent possible post-operative complications. Rehabilitation begins with breathing exercises and then lower limb exercises are introduced: foot ankle circles, alternating dorsal and sole flexion of the foot. In order to facilitate the outflow of blood and lymph towards the heart, it is advisable to perform these exercises in such a way that the limb being exercised is slightly raised above body level.

An important element in women undergoing surgery is introducing physioprophylaxis and anticoagulant treatment as soon as possible. Compression therapy is one of the methods used to reduce venous stasis. It is applied along the entire length of the lower limb, with the greatest compression applied in the toe area and with a gradual reduction in compression towards the proximal limb [50]. During bandaging, the limb should be elevated above body level. Thoracic track breathing exercises are also used. Prior to surgery, it is also important to educate patients on the ability to cough up effectively and expectoration, with particular attention to stabilising the post-operative wound [50].

Regardless of the type of operation, it is important to treat the scar as swiftly as possible in order to reduce the risk of scarring and to provide careful instruction on scar management at home. A post-operative wound is generally tended with an intradermal suture, which can remain in place until absorption or, in the case of a non-absorbable suture, it is removed on the fifth post-operative day. Once the scab has fallen off, scar therapy can begin. Ointments containing substances that reduce the inflammatory reaction and compounds with a lytic effect are most often used as support. Manual techniques and selected massage techniques can be used to mobilize the scar [51,52]. This treatment can be started when the sutures have been removed and the wound is healed. First, stroking and rubbing techniques are used, which we also teach patients to perform at home on their own. The whole scar should be worked on by successive circular movements in place, changing their direction in a cyclic manner. When the scar is already soft and does not hurt, it is advisable to activate the deep adhesions by separating these adhesions from the subcutaneous tissue, rubbing them by first making circular movements and then movements in opposite directions. When performing massage techniques, the patient can use ointments and gels recommended by doctors to lubricate scars, which in turn will benefit the regeneration process and enhance the physiotherapeutic effect [51,52].

In the case of laparoscopic surgery, special attention should be paid to activating the abdominal muscles as little as possible, while at the same time not forgetting to adopt a vertical position and further rehabilitation management [53].

If there are no medical contraindications, physiotherapy is started on the first postoperative day. In the case of a laparotomy, the patient changes positions from lying on her back to lying on her side and vice versa, with her lower limbs bent at the hip and knee joints (and thus her abdominal muscles relaxed) by performing a rotation of her entire torso. Once a sitting position is reached, upright positioning is introduced.

It is also important to make patients aware of the importance of exercise and physical activity at every stage of endometriosis treatment. The symptoms associated with endometriosis stem from a local inflammatory reaction in the peritoneum caused by ecotopic endometrial tissue.

Regular exercise has been shown to have a protective effect against diseases that include inflammatory processes, as regular exercise involves a systemic increase in anti-inflammatory and antioxidant cytokines and a decrease in oestrogen levels in endometrial cells [54]. Systematic physical exercise stimulates the effect of reducing the menstrual blood flow, regulating both ovarian function and oestrogen levels. Regular amateur physical activity can decrease oestrogen levels and reduce the bioavailability of this hormone [55,56]. A significant reduction in the risk of developing endometrial cysts has been observed in patients doing high-intensity exercise at MET 6 levels [57,58]. Patients should be taught that healthy habits are not only a matter of physical activity, but also mental health and education, which facilitate a healthy lifestyle. The most common forms of activity are yoga, jogging and exercise [59].

Physiotherapy as a complementary treatment for women with symptomatic endometriosis has significant benefits in reducing pain, and although there are no conclusive study findings, many women indicate this type of therapy as being effective.

Physiotherapeutic work in women with endometriosis can be complemented successfully by physiotherapeutic interventions, including manual techniques [60] (Table 2), which can be effective in relieving pain occurring in the pelvic area.

Pelvic organ pain is often perceived as somatic pain, due to innervation from the same level, which is associated with peripheral sensitization [61,62,63,64,65,66].

Visceral manual techniques, proposed by Jean-Pierre Barral [67], deserve special attention for manual work. Visceral manual therapy encompasses the three-dimensional dynamics of body biomechanics: musculoskeletal structure, musculo-fascial structure, connective tissue and organs, and reflex activity in the central and peripheral nervous system, as well as the circulation and drainage of fluid systems in the human body [67]. The aim of manual peritoneal therapy for dysfunction in the female reproductive system is to promote movement, articulation and improvements in tissue rhythm, which is a physiological phenomenon and a fundamental aspect of life. Treatment of dysfunction in the reproductive system involves assessing the abdominal and pelvic cavity. In order for this cavity to be able to maintain physiological movement, the organs must move in relation to each other and also in relation to the membranes that surround them. There are three pathomechanisms that interfere with the sliding movement between the organs and the surrounding musculo-fascial structures and which can lead to pain and other dysfunctions. These are referred to as pain, changes in local tissue dynamics and central sensitization [63]. The general management of reproductive system dysfunction involves restoring postural balance, breathing, pelvic activity and balancing the pressures between the different diaphragms in the body. It is also important to pay attention to muscle tone in the pelvis and thoracolumbar fascia, centralisation of the hip joint, sacroiliac region and the symphysis pubis [68]. Therefore, visceral manual therapy should begin with a postural analysis, as mobility abnormalities in the lumbar spine, hip joints and symphysis pubis are key contributors to muscle-fascial tension dissonance [69,70,71]. Assessment of the spine is also important, particularly in the thoracic region at the level of the 12th vertebra and the lumbar region at the level of the 1st lumbar vertebra and at the lumbosacral transition and the sacroiliac joint. Restrictions due to sympathetic innervation at the thoracolumbar spine can lead to vasodilation in the small pelvis. Blood pressure is lowered, and oxygen and nutrient supply is significantly impaired [66].

Uterine palpation allows the presence of adhesions, the shape, mobility and position of the uterus to be determined. The physiological state of the uterus can be comprehensively assessed when there is no strong resistance or pain in the lower abdomen. The uterus should be flexible to some extent. A strong response to pressure may signal adhesions. A complete lack of resistance may indicate uterine retroversion [41,43]. Uterine relaxation techniques are useful in the treatment of reduced uterine mobility. Additionally, these techniques aid uterine vascular drainage [67,70].

Myofascial trigger points (MTrPs) can occur in the vagina, urethra or rectum; these are small, palpable, hypersensitive nodules that can either be active or latent [72,73]. Transvaginal techniques can be used to deactivate such trigger points.

Patients with endometriosis report that the disease affects all aspects of a woman’s physical and psychological health, hence it is important that support is multifaceted.

Contraindications for physiotherapy in patients with endometriosis include malignant tumours of the reproductive organs prior to 12 months after completion of surgical therapy, radiotherapy or chemotherapy excluding hormonal therapy, acute inflammation within the reproductive organs, acute bacterial infections, fungal infections established large myomas qualifying for surgical treatment unexplained bleeding from the reproductive tract, and pregnancy.

Physiotherapy is safe and effective and can make a significant difference to the symptoms associated with pelvic dysfunction and therefore to women’s quality of life.

## 4. Limitations

Despite the positive impact of physiotherapy resulting from its practical use in women with endometriosis, there is a lack of scientific publications of a research nature, with large study groups and with a well-described methodology for using the different forms of physiotherapy. Certainly, confirmation of practice through science would make it possible to indicate which form of physiotherapy may be most effective in the treatment of endometriosis.

## 5. Conclusions

The use of physiotherapy and its forms as an adjunctive treatment for endometriosis requires scientific research to test the efficacy of physiotherapy and determine which form will be the best to improve women’s biopsychophysical condition. Due to the small number of publications, it seems that the use of physiotherapy in the treatment of endometriosis is underestimated and underpublicised.

This review provides information that the following are effective treatments to reduce pain and improve quality of life for women with endometriosis: pulsed high-intensity laser therapy, transcutaneous electrical nerve stimulation (TENS), pulsed electromagnetic fields and manual physiotherapy.

## Figures and Tables

**Table 1:** Form of physiotherapy applied, e.g., laser therapy, TENS, electromagnetic fields.

Study	Treatments	Materials and Methods	Clinical Benefits
Thabet et al., 2020 [35]	Pulsed high-intensity laser therapy three times per week for 8 weeks. A wavelength of 1064 nm, a fluency level of 510–1780 mJ/cm^2^, a brief duration of 120–150 ls, a low frequency of 10–30 Hz, 0.1 percent duty cycle, a probe diameter of 0.5 cm, and a spot size of 0.2 cm	The sample included 40 women aged between 24 and 32 years with endometriosis of a mild or a moderate degree. They were randomly assigned to two groups, group 1 of 20 women received pulsed high-intensity laser therapy three times per week for 8 weeks, as well as the usual regimen of hormonal treatment given to endometriosis patients, and group 2 of 20 women received sham laser treatment three times per week for 8 weeks and the usual regimen of hormonal treatment. For all patients, pain, the degree of endometriosis, and quality of life were measured using present pain intensity and pain relief scales, laparoscopy, and the Endometriosis Health Profile (EHP-5) before treatment began and at the end of the 8 weeks.	In comparison to the sham laser treatment, pulsed high-intensity laser therapy produced a significantly different result in women with endometriosis. Pulsed high-intensity laser therapy is an effective method of pain alleviation, reducing adhesions, and improving the quality of life in women with endometriosis.
Mira et al., 2020 [36]	Applied TENS and hormonal therapy for 8 weeks in the S3–S4 region, 30 min session	Included a hundred and one participants with DIE in electrotherapy (n = 53) (hormonal treatment + electrotherapy) or control group (n = 48) (only hormonal treatment) for 8 weeks of follow-up. The primary measurement was chronic pelvic pain (CPP) using a visual analogue scale (VAS) and deep dyspareunia. The secondary outcomes were the quality of life measured using the endometriosis health profile (EHP-30) and sexual function using the female sexual function index (FSFI).	Alleviation of CPP was observed only in the electrotherapy group. In terms of profound dyspareunia, an improvement was observed for both groups. Considering the secondary outcomes, a higher post-treatment total score for EHP-30 was observed in both groups. With regard to sexual function, a statistically significant improvement in the FSFI score was observed in the electrotherapy group, with an increase in scores in the domains of lube and pain.
Mira et al., 2015 [37]	Applied TENS for 8 weeks in the S3–S4 region for both groups, 30 min session	22 women with deep endometriosis diagnosed in the culdesac and intestinal loop who sustained pelvic pain and/or deep dyspareunia, despite continuous clinical medication. Participants received intervention and were randomized into two groups: Group 1: acupuncture-like TENS (Dualpex 9611) (n = 11) and Group 2: self-applied TENS (Tanyx1) (n = 11). All women had been undergoing hormone therapy with continuous progestin alone or combined oral contraceptives for at least three months, reporting pelvic pain and/or deep dyspareunia persistence, associated or not with other pain complaints (dysmenorrhea, dyschezia and dysuria).	Both resources (acupuncture-like TENS and self-applied TENS) demonstrated effectiveness as a complementary treatment of pelvic pain and deep dyspareunia, improving quality of life in women with deep endometriosis regardless of the device used for treatment.
Jorgsen et al., 1994 [40]	Pulsed ElectroMagnetic Fields one time per week for one month, 15–20 min.	Short exposures of affected areas to the application of a magnetic induction device producing short, sharp, magnetic-field pulses of a minimal amplitude to initiate the electrochemical phenomenon of electroporation within a 25 cm^2^ focal area.	Of the 17 patients presenting with a total of 20 episodes of pelvic pain, of which 11 episodes were acute, seven chronic and two acute as well as chronic, 16 patients representing 18 episodes (90%) experienced clear relief, while two patients represented two episodes reported incomplete pain relief.

**Table 2:** Application of non-invasive site-specific manual physiotherapy techniques.

Study	Treatments	Materials and Methods	Clinical Benefits
Wurn et al., 2011 [60]	To assess the efficacy of a non-invasive, site-specific manual physiotherapeutic technique in ameliorating dyspareunia and dysmenorrhea, commonly associated with endometriosis, by performing a retrospective and prospective analysis, respectively.	For Study I (Retrospective analysis of the effect on dyspareunia in women with endometriosis) 14 female patients surgically diagnosed with endometriosis (out of 23 previous participants of a previously conducted Sexual Function Study) (13) were enrolled. A total of 18 female subjects were enrolled for Study II (Prospective analysis of the effect on dyspareunia and dysmenorrhea in women with endometriosis). Human female subjects, all surgically diagnosed with endometriosis, were enrolled in each of the studies after informed consent. Each subject underwent 20 h of site-specific manual physical therapy (Wurn Technique) designed to address adhesions and restrictions in soft tissue mobility in the abdomen and the pelvic floor. A post-test was completed 6 weeks after treatment.	Site-specific manual physiotherapy might offer a non-pharmacologic and non-surgical alternative in the treatment of dyspareunia and dysmenorrhea in endometriosis patients. Evaluation incorporated an outcome prediction based on the Female Sexual Function Index (FSFI) for analysing the effect on dyspareunia and sexual function (n = 14) and quantitative differences in ratings of average pain during menstrual cycle and intercourse based on the Mankoski Pain Scale for analysing the effect on dysmenorrhea and dyspareunia (n = 18), respectively. Data were analysed using the Wilcoxon signed-rank test (two-sided)
